# Food Safety Practice and Associated Factors among Meat Handlers in Gondar Town: A Cross-Sectional Study

**DOI:** 10.1155/2020/7421745

**Published:** 2020-02-24

**Authors:** Dawit Getachew Yenealem, Walelegn Worku Yallew, Shafi Abdulmajid

**Affiliations:** ^1^Department of Environmental and Occupational Health and Safety, Institute of Public Health, College of Medicine and Health Sciences, University of Gondar, Gondar, Ethiopia; ^2^Department of Public Health, Eastern African Collage, Harrar Campus, Harrar, Ethiopia

## Abstract

*Introduction*. Animal sources of foods (ASF), including meat, are a source of high-quality nutrients. However, meat composition makes it an ideal medium for the growth of a good number of microorganisms. Around 600 million foodborne illnesses and 420000 deaths occur each year due to poor food handling practice. Thus, probing into meat handling practice will be an insatiable input for the intervention. This study aims to investigate the level of meat handling practice and associated factors among meat handlers in butcheries in Gondar town, Ethiopia.

**Method:**

The study was a community-based cross-sectional study among butcher shops in Gondar town from April 20 to 30, 2019. Data were collected using a pretested structured questionnaire by trained data collectors among 214 meat handlers from butcher shops. Multivariable logistic regression analysis with a 95% confidence interval (CI) was used to identify the factors significantly associated with a good level of meat handling practice.

**Result:**

More than half of the meat handlers 66.4% (95% CI: (59.8, 72.4)) in butcher shops had a good level of meat handling practice. Level of attitude (AOR = 4.45; 95% CI, 2.09–9.43) and knowledge (AOR = 2.04; 95% CI, 1.09–3.82) were significantly associated with a good level of meat handling practice. The majority of respondents wash their hands after disposing garbage (91.6%) with less vigilance after smoking, sneezing, or coughing (64.0%).

**Conclusion:**

The study revealed that the level of food handling practice was unsatisfactory among meat handlers. This result is a testimony to the prevailing potential risk faced by consumers due to the disregarding of hygienic behaviors by food handlers. Considering attitude and knowledge are associated with the outcome variable, investing time on behavioral change activities that will contribute to the improvement of meat handler's attitude & practice, primarily focusing on reducing working while ill is essential. Therefore, much supervisory and coaching work will be expected from local health departments & regulatory bodies.

## 1. Introduction

Most fresh food, particularly those from animals, are highly vulnerable to microbial contamination and food poisoning [1]. Meat composition makes it an ideal medium for the growth of a good number of microorganisms [2] due to richness in nutrients [3]. The majority of foodborne diseases arise from the food of animal origin [4, 5]. The food handler's health status and hygiene practice are the foremost determinants of food contamination [6]. Food poisoning happens as a result of ingesting food contaminated with microorganisms or their toxins, the contamination springing up from insufficient protection methods, unhygienic dealing with practices, cross-contamination from food contact surfaces, or men and women harboring microbes [7]. This can result in quality deterioration and, hence, quantity losses, economic losses, and public health concerns [8, 9].

According to the World Health Organization, almost 1 in 10 people fall ill, and 420 000 die every year, dining on cuisine tainted by microorganisms [10]. Other reports depicted that around 600 million foodborne illnesses and 420,000 deaths occur each year due to poor food handling practice [11], in which substantial proportion goes to meat-related hazards [12].In the Netherlands, About 53% of the foodborne burden and 31% of all food-related cases were associated with meat [12]. A World Bank study finds that the impact of unsafe food costs low- and middle-income economies, nearly US$ 110 billion in decreasing productivity and medical expenses each year [13]. One Ethiopian study claims that contamination of beef while transferring from the abattoir to the butcher shops with the highest source of contamination attributed to abattoir workers [14]. Foodborne diseases have been increasing in recent years, with an enormous impact on the health and economy of developing countries than those of developed countries [7, 15]. The impediment of socioeconomic development has manifested this by straining the health-care system and harming national economies, tourism, and trade [16].

Food handling and hygiene have been a great concern across industries [17]. Foods can be mishandled during preparation, processing, or storage [18]. It has been shown that most outbreaks of food poisoning result from improper food handling practices [19] that implicate the food handlers [20]. According to the Codex Alimentarius Commission, improper food handling is a major cause of foodborne diseases, and poor hand hygiene is a significant risk factor in the occurrence of food contamination [21]. Mishandling food supposed to be implicated in 97% of all food-borne illnesses associated with food service establishments [22]. European Food Safety Authority [23] reports that around 48.7% of foodborne illnesses are associated with food services in the food premises [21]. This was underlined by the fact that mishandling [24], worker health and hygiene [25], and the presence of *Escherichia coli* and *Staphylococcus aureus* on the hands of food handlers [26] were stated as specific reasons in studies. In developing countries, Foodborne diseases occur because of poor food handling and sanitation problems [27]. Food workers in many settings have been responsible for food borne disease outbreaks [28], and there is no indication that this is diminishing [19].

Thus, probing in handling practice of meat handlers will be an insatiable input for the intervention. Three factors are playing a fundamental role in food poisoning outbreaks concerning food handlers: knowledge, attitude, and practice [29]. Because meat is a highly perishable food, the knowledge and level of training of meat handlers in the meat industry in hygiene and food safety are of particular importance in ensuring the health and safety of the consumer [30]. Practices will render antecedently uncontaminated foods unsafe to eat, e.g., through cross contamination, and contaminated foods safe to eat, e.g., through thorough preparation [31]. The informal methods of meat handling and marketing meat by butcheries undermine meat quality and safety [32], which is shared by the similar socioeconomic conditions of Ethiopia. Customarily, food is sold and displayed in open shops without proper guarding [33], and cold-chain process [14] of hanging raw meat on the hooks and offering for consumption to consumers were followed in butcheries. Food-borne disease outbreaks remain a major global health challenge, and cross contamination from raw meat due to poor handling is a major cause in developing countries [19]. In Ethiopia, despite rising prices, the demand for meat products is dramatically increasing from 7.0 kg in 2000 to 8.5 kg in 2009 [34]; and the consumption of raw meat becomes a symbol of status [7].This study aims to investigate the level of meat handling practice and associated factors among food handlers in butcheries across Gondar town. It will be a spring board for further inquires on the problem and valuable inputs for concerned regulatory, health promotion bodies.

## 2. Methods

### 2.1. Study Design, Setting, and Period

The study was a community-based cross-sectional study among butcher shops in Gondar town from April 2019. The town of Gondar is located north of Addis Abeba at 737 km and 265 km from the regional capital Bahir Dar. Gondar was founded in 1635 and located in the central Gondar Zone of the Amhara Region, and it is one of the tourist destination places which have been a seat of a government. According to the central statistical agency of Ethiopia, in 2017, Gondar projected to be home for 360,600 people [35]. Administratively, the town is divided into 12 administrative areas. During the study, there were reportedly about 146 butcher shops in Gondar town. Among them, 129 are Christian ones, and the rest are Islamic ones.

### 2.2. Inclusion and Exclusion Criteria

Meat handlers who have direct contact with meat and meat handling surfaces with one year experience were considered to study population. Furthermore, those who were randomly selected were a study population. Those meat handlers who are not well communicated due to any disability or illness and seriously ill workers were excluded from the study.

### 2.3. Sample Size Determination and Sampling Procedure

The sample size was determined using a single population proportion formula *n*=*Z*^2^*p*(1 − *p*)/*d*^2^. Since similar studies in our country on this study subjects are lacking, we take 50% prevalence for meat handling practice with 95% confidence interval and margin of error 5% between the sample and the underlying population, which give us a sample size of 384. However, the final sample size was determined by using correction formula *FN*=*n*/1+*n*/*N* (since *N* < 10,000).By adding 10% nonresponse rate, the sample sizes became 224.

The community-based cross-sectional study design was conducted. A systematic random sampling technique was used to select each butcher shop. Systematic random sampling was followed by proportional allocation for those groups of workers more than one member within butcher shops. Two hundred and fourteen meat handlers were selected from 12 subcities by systematic random sampling methods since we are unable to find a list of our subjects.

### 2.4. Operational Definition

#### 2.4.1. Meat Handling Practice Level

The respondents who scored less than 70% of the correct answer of their response to 20 meat handling-related practice questions were considered as having “poor level of practice,” and those who scored higher than or equal to 70% were considered as having “good practice level” [15].

#### 2.4.2. Meat Handling Knowledge Level

The respondents who scored less than 70% of the correct answer of their response to 20 meat handling related knowledge questions were considered as having “poor level of knowledge” those who scored higher than or equal to 70% were considered as having “good level of knowledge” [7, 15, 20].

#### 2.4.3. Meat Handling Attitude Level

The respondents who scored less than 70% of the correct answer of their response to 20 meat handling-related attitude questions were considered as having “poor level of attitude,” and those who scored higher than or equal to 70% were considered as having “good level of attitude” [7, 15, 20].

### 2.5. Data Collection Tools and Procedures

Data were collected from meat handlers working in butcher shops in Gondar town. An interview-administered data collection technique was used to collect data. Two environmental health professionals were deployed for supervision, along with four environmental and occupational health and safety (EOHS) final year students as data collectors. The questionnaire was initially prepared in the English version, then translated to Amharic, and back to English to check consistency and clarity of the question. A pretested Interviewer-based structured questionnaire adapted from different literature [7, 15, 19–21, 24] to fit with both subject area, and local context was used to collect data in which a similar version was used in Ethiopia [20]. The questionnaires comprised four blocks of sections, namely: sociodemographic, knowledge, and attitude, and practice questions.

In the knowledge part, 20 close-ended questions were focusing on personal hygiene, cross contamination, microbiological food hazards of specific foodborne diseases, safe food handling, person-to-person food-borne disease transmission, and handwashing knowledge. Each question was followed by three potential responses (i.e., yes, no, and I don't know).

The subsequent part of the questionnaire was inquired about the attitudes of the responders by forwarding questions related to various hygienic measures for food safety. It comprises twenty questions where food handlers were asked to indicate their level of agreement to the statements using a three-point rating scale (i.e., I agree, I don't agree, and I don't know). The third option, “I don't know” has been introduced to facilitate ease of responding for participants by complimenting for thoughts characterized by an *Undecidedness*, *uncertainty*, or not aware of the inquiry at all.

The last section assesses practices of food workers by their selfreported hygienic behaviors. Similar numbers of questions were provided with Yes or No options. The items include eating or drinking at the workplace, washing hands: after handling waste, using the toilet, coughing or sneezing; habit of wearing gloves, masks & aprons during work; routines during having illness and even presenting with cuts, wounds, bruises or injuries on hands; wear jewelry when handling meat. Response to part two to three treated as categorical variable considering correct answer as 1 and incorrect answer as 0 (including I don't know). In addition, a scale ranging between 0 and 20, which represents the total number of questions on the three sections were considered.

### 2.6. Data Quality Management and Analysis

All the questionnaires were checked visually, coded, and entered into Epi Info software version 7 and exported into SPSS version 20.0 for further analysis. Descriptive statistics were computed to summarize data, including frequenciesand percentages, and mean for each item of the questionnaire was presented by using graphs, tables, and charts. Moreover, tabulation and different forms of graphs were used to present the result. Bivariable and multivariable logistic regression analysis was conducted to identify factors associated with Meat handling practice. The strength of the associated factors was presented by the odds ratio with 95% CI. A *p* value of less than 0.05 was considered statistically significant. Compiled results were presented in the form of text, tables, or graphs.

Every day, during the collection of data, each completed questionnaire was checked for consistency and completeness by investigators. Through the course of data collections, a regular discussion was held in between group members in which problematic issues arising during data collection, and incomplete data was cleared.

## 3. Results

### 3.1. Sociodemographic Characteristics

The response rate becomes 95.53%. Mean age (±SD) of study participants become 27.22 ± 4.82 years. Among the participants, most are male (77.6%), and the young were aged twenty-seven or below (52.8%). One-third of respondents attend primary education, 33.6%. The mean monthly income of a respondent is 2046.06 ETB. One hundred and ninety-seven (92.1%) describes their employment status as permanent (Table 1).

### 3.2. Meat Handling Practice Responses of Participants

Most of the respondents are good at safety practices such as washing hands after disposing of garbage and before handling meat (91.6% & 92.1%). However, they are less serious about washing hands after visiting the toilet; and to the worst, after sneezing & coughing. Nearly half of them remove personal stuff while processing meat (51.4%). A considerable proportion of participant does not refrain from handling meat while felt ill or having cut, wounds in their hands. The lowest right answer is given to the practice of using gloves (9.8%) (Table 2).

### 3.3. Factors Associated with Meat Handling Practice

Level of practice becomes 66.4% [95% CI: (59.8, 72.4)]. Sex, religion experience, attitude, and practice were found significantly associated with a value of *p* value of <0.2 in bivariable logistic regression. While running into multivariable logistic, attitude and knowledge remains significant (Table 3).

## 4. Discussion

Maintenance of proper hygienic practices is at the top of the agenda in food & drinking establishments, while handling is essential to provide fresh and healthy meat for public consumption [36]. In this cross-sectional study, we try to explore the level of good practice among butcher houses with potential determinants. The level of good practice of meat handling among workers in butcher houses becomes 66.4%. This is the reality of developing countries like Ethiopia, where either awareness or lack of essential facilities does not make practice as expected. It is similar to food handlers in food establishments in Dessie (72%) [37], Dangila (52.5%) [38], Ethiopia.

It is lower than some of the hygiene behavior-related responses like washing hands after using the toilet in Malaysian canteens 75.4% [39], Ethio-Somalia butchers 86.8% [20]; after handling garbage (94.3%), smoking and sneezing 95.4%, respectively [21], refraining from dining (70.7%) or smoking (96.8%) at the workplace [19]. Similarly, utilization of PPE-related practices include hairnet use in Saudi 96.6% [21] and wearing an apron in Iran 95.9% [19]. The discrepancy will be due to sociodevelopment, enforcement capacity, and even Islamic religious routines that influence personal hygiene conducts due to their stringent rules about the purity of foods in food handling [40].

It is higher than mothers in Debarq town [41], public food establishments in northwest Ethiopia [42], and Araba minch [43]. Moreover,it is higher, compared to unaggregated responses like handle money while receiving customers in Gondar butcher shops (45.3%) [9], wearing an apron during work (38.3%) [7]. The difference can be implicated to the very variation in analysis plan, cutoff point, operational definition, and scope of research and tool composition.

Those workers with a good level of knowledge concerning food safety have odds of 2 times more engaged in good practices. It is accepted that knowledge alone is insufficient to trigger preventive practices, and some mechanism is needed to motivate action and generate positive attitudes [22]. This is in line with Malaysian food handlers (*p* = 0.041) [44], eastern Ethiopia (AOR = 10.4, 95% CI: (4.6, 23.81)) [45]. The educational level appears to affect the attitude (*p* = 0.001) and practice (*p* = 0.009) of the participants [46] that shows an indirect influence of knowledge to practice; having an educational level plays an undisputed role in shaping the knowledge of food handlers.

However, a significant negative association was reported between knowledge and practices (*p*=0.04) in Iran meat processing industry [19]. This does not necessarily imply that nothing is contributed by knowledge; rather, it indicates some impediments that prevent the conversion of knowledge into attitude and gradually to tangible practices. These bottlenecks can be like unavailability of bathroom facilities [45] and intermittent water supply.

Attitude manifests itself as the strongest factor that steers the level of practice of food handlers. The odds of exercising a good level of meat handling practice are 4.5 times among those with a good attitude towards safe food handling practice. Attitude is the proximal factor that determines the translation into observable action. This supported with previous investigations like public food handling establishments in northwest Ethiopia (AOR = 1.97, 95% CI 1.04, 3.72) [42], Malaysian food handlers (*p* = 0.041) [44], and Debarq mothers (AOR 3.67, 95% CI: 2.27, 5.94) [41]. A discordant result was observed in some literature; for example, Iranian study came up with a significant negative correlation between attitudes and practices (rs = _0.27, *p* = 0.009) [19]. This may be attributed to scenarios in which the attitudinal change is just due to social desirability bias.

The study is in short of incorporating observations during meat handling operations. Thus, it is a result of a self-report of the meat handlers. Moreover, scarcity of the related literature gives a hard time to compare results with aggregate findings rather than resorting to using specific response questions. It is crucial to admit that having a relatively small sample size will have a role in overall generalizability.

## 5. Conclusion

More than half the number of meat handlers in Gondar town had a good level of self-reported food safety practice. In general, their food safety practice level was found comparable with earlier studies and by far lower than universally expected a higher standard of practice. There was no significant relationship with the level of food safety practice and some demographic variables (age, educational level, income, and experience). Knowledge and attitude were found as the factors having a stronger significant association with the food safety practice of meat handlers. Therefore, food safety knowledge and positive attitude of meat handlers, through frequent mentoring-based supervisory work, facilitating the availability of sanitation facilities are important interventions to enhance their level of food safety practice.

## Figures and Tables

**Table 1:** Sociodemographic and behavioral characteristics of meat handlers working at butcher shops at Gondar town, April 2019 (*n* = 214).

Variables	Meat handlers	
	Frequency	Percentage
Sex		
Male	166	77.6
Female	48	22.4
Age		
≤27	113	52.8
≥28	101	47.2
Religion		
Orthodox	175	81.8
Others^¥^	39	18.2
Educational status		
Unable to read and write	19	8.9
Primary education (1–8)	72	33.6
Secondary education (9–12)	76	35.5
Higher education (12+)	47	22.0
Marital status		
Married	94	43.9
Single	120	56.1
Income		
1000 and below	46	21.5
1001–2000	101	47.2
2001–3000	31	14.5
3001 and above	36	16.8
Working condition		
Permanent	197	92.1
Contract/daily	17	7.9
Knowledge		
Poor	108	50.5
Good	106	49.5
Attitude		
Poor	41	19.2
Good	173	80.8

NB: ^¥^Muslim & protestant.

**Table 2:** Meat handling practice of meat handlers at butcher shops at Gondar town, April 2019 (*n* = 214).

Practice questions	Responses *n* (%)	
	Right	Wrong
Do you eat or drink at your workplace?	107 (50.0)	107 (50.0)
Do you smoke inside meat processing areas?	194 (90.7)	20 (9.3)
Do you use gloves while handling meat?	20 (9.3)	194 (90.7)
Do you handle money while processing meat?	21 (9.8)	193 (90.2)
Do you wash your hands before and after handling meat?	197 (92.1)	17 (7.9)
Do you wash hands after handling waste/garbage?	196 (91.6)	18 (8.4)
Do you wash hands after using the toilet?	192 (89.7)	22 (10.3)
Do you wash your hand after smoking, sneezing, or coughing?	137 (64.0)	77 (36.0)
Do you wear an apron while working?	101 (47.2)	113 (52.8)
Do you wash your aprons after each day's work?	175 (81.8)	39 (18.2)
Do you wear a mask while working?	81 (37.9)	133 (62.1)
Do you wear a hairnet or a cap while working?	198 (92.5)	16 (7.5)
Do you use and paint nail polish when handling meat.	190 (88.8)	24 (11.2)
Do you properly clean the meat storage area before storing new products?	199 (93.0)	15 (7.0)
Do you use the sanitizer when washing service utensils (knives, hooks, and cutting boards)?	160 (74.8)	54 (25.2)
Do you replace knives or sterilize them after each meat processing?	135 (63.1)	79 (36.9)
Do you remove your work equipment when using the toilets?	188 (87.9)	26 (12.1)
Do you remove your personal stuff such as rings, necklaces, watch, etc. while processing meat	110 (51.4)	104 (48.6)
Do you handle/process meat when you are ill?	116 (54.2)	98 (45.8)
Do you handle/process meat when you have cuts, wounds, bruises, or injuries on your hands?	140 (65.4)	74 (34.6)

**Table 3:** Bivariable and multivariable logistic regression of factors associated with the practice of meat handlers working at butcher shops at Gondar town, April 2019 (*n* = 214).

Variables	Meat safety practice		COR (95% CI)	AOR (95% CI)
	Good	Poor		
Experiences				
2 and below	65	29	1	1
3-4	52	25	0.92 (0.48, 1.77)	0.93 (0.46, 1.87)
5 and above	25	18	0.62 (0.29, 1.30)	0.62 (0.28, 1.41)
Sex				
Male	106	60	1	1
Female	36	12	1.69 (0.82, 3.51)	2.03 (0.91, 4.50)
Religion				
Orthodox	120	55	1.68 (0.83, 3.42)	1.53 (0.71, 3.33)
Others^¥^	22	17	1	1
Attitude				
Good	127	46	4.78 (2.33, 9.82)^*∗∗∗*^	4.45 (2.09, 9.43)^*∗∗∗*^
Poor	15	26	1	1
Knowledge				
Good	80	26	2.28 (1.27, 4.04)^*∗∗*^	2.04 (1.09, 3.82)^*∗*^
Poor	62	46	1	1

NB: significant at *p*^*∗*^=0.05, ^*∗∗*^=0.006, ^*∗∗∗*^=0.0001 and 1 is the reference; ^¥^ **=** Muslim and protestant.

## Data Availability

The data used to support the findings of this study are available from the corresponding author upon a reasonable request.

## Abbreviations

AOR: Adjusted odds ratio
CI: Confidence interval
COR: Crude odds ratio
ETB: Ethiopian Birr
Km: Kilometer
SD: Standard deviation
SPSS: Statistical package for social sciences
PEP: Personal protective equipment.
